# circCELSR1 facilitates ovarian cancer proliferation and metastasis by sponging miR-598 to activate BRD4 signals

**DOI:** 10.1186/s10020-020-00194-y

**Published:** 2020-07-08

**Authors:** Xiang-Yang Zeng, Jing Yuan, Chen Wang, Da Zeng, Jia-Hui Yong, Xiao-Yan Jiang, Hua Lan, Song-Shu Xiao

**Affiliations:** Department of Gynecology, the Third Xiangya Hospital of Central South University, No.138 Tongzipo Road, Changsha, 410013 Hunan Province PR China

**Keywords:** circCELSR1, miR-598, BRD4, Ovarian cancer, Tumor metastasis

## Abstract

**Background:**

Ovarian cancer is one of the most common gynecologic cancers and has high mortality rate due to the lack of early diagnosis method and efficient therapeutic agents. circCELSR1 is up-regulated in ovarian cancer, but its role and mechanisms in ovarian cancer are unclear.

**Methods:**

Gene expression of circCELSR1, miR-598 and BRD4 in ovarian cells was examined by qRT-PCR. Protein level was determined by Western blotting. Bioinformatic analysis and luciferase assay determined the molecular binding among circCELSR1, miR-598 and BRD4 3′ UTR. Cell proliferation, migration, invasion and apoptosis were determined by colony formation, wound healing assay, transwell assay and flow cytometry analysis, respectively. An abdominal cavity metastasis nude mice model was used to determine the in vivo function of circCELSR1.

**Results:**

circCELSR1 and BRD4 were promoted, but miR-598 was suppressed in various ovarian cancer cells. circCELSR1 bound to miR-598 and promoted expression of its downstream target BRD4. Knockdown of circCELSR1 suppressed proliferation, migration, invasion and epithelial-mesenchymal transition (EMT), but promoted apoptosis in ovarian cancer cells, and these effects were reversed by miR-598 inhibition or BRD4 overexpression. circCELSR1 inhibition decreased the expression of BRD4 and its downstream proliferation/migration related genes by targeting miR-598. Furthermore, knockdown of circCELSR1 suppressed ovarian cancer growth and metastasis in nude mice.

**Conclusion:**

Knockdown of circCELSR1 inhibited BRD4-mediated proliferation/migration related signaling via sponging miR-598, thereby repressing ovarian cancer progression. This study provides a new regulatory mechanism of ovarian cancer may facilitate the development of therapeutic agents for ovarian cancer.

## Background

As one of the most common gynecologic cancers, ovarian cancer has the highest mortality rate and has become a global concern. Estimated that there were 22,240 new cases of ovarian cancers diagnosed and 14,070 deaths in 2018 (Siegel et al. 2018). Ovarian cancer is typically diagnosed at a late stage and has no effective screening strategy for early diagnosis (Sundar et al. 2015). Although incremental advances in the treatment have been made, there is still no efficient therapeutic agents for the treatment of ovarian cancer to date (Doo et al. 2019). Additionally, due to the metastasis of ovarian cancer cells, leading to a result that the 5-year survival rate is still no more than 30% (Yeung et al. 2015). Hence, investigations on the molecular mechanism of ovarian cancer metastasis may contribute to the development of effective therapeutic agents for ovarian cancer treatment.

Circular RNAs (CircRNAs) are a group of non-coding RNAs without the free 3′ and 5′ ends and form the final continuous loop RNAs (Santer et al. 2019). The first identification of circRNAs was at about 20 years ago, however the investigations on circRNAs in the regulation of cellular activities became prevalent recently (Ebbesen et al. 2016). More and more evidence revealed that circRNAs are crucial in the regulation of tumorigenesis and invasiveness for various cancers (Zhao and Shen 2017). Typically, circRNAs regulate gene expression via the post-transcription process, normally by an RNA-RNA interaction to miRNAs. It has been demonstrated that circRNAs act as competing endogenous RNA (ceRNA) and bind to miRNAs to block their function (Cheng et al. 2015). The role of circRNAs in regulating ovarian cancer has been investigated recently, however, the total amount of studies is still limited. Until recently, circRNAswere found to regulate the proliferation and metastasis (Zhao et al. 2019) and invasion (Karedath et al. 2019) of ovarian cancer cells, and affect epithelial mesenchymal transition (EMT) (Zhang et al. 2019) of ovarian cancer cells. Recent studies revealed that circCELSR1 was significantly up-regulated in ovarian cancer (Ning et al. 2018). However, the role and mechanisms of circCELSR1 in ovarian cancer remain unclear.

MicroRNAs (miRNAs) is a kind of non-coding endogenous RNAs of 21–25 nucleotides (nts) in length (Mohr and Mott 2015). Accumulated evidence indicated that miRNAs plays central role in regulating various cell activities, such as tumorigenesis (Yonemori et al. 2017). miR-598 was a recent discovered miRNA that was considered to play a role in different kinds of tumor progression. For example, miR-598 was found to inhibit cell proliferation and invasion of glioblastoma cells (Wang et al. 2018). miR-598 suppressed migration and invasion of non-small cell lung cancer cells via inhibiting Derin-1 and EMT (Yang et al. 2018). Additionally, miR-598 exerted suppressive effects for proliferation and invasion of non-small cell lung cancer cells by targeting ZEB2 (Tong et al. 2018). The reports about the role of miR-598 in regulating ovarian cancer are very limited. To date, there was only one report indicating that miR-598 inhibited proliferation and metastasis of ovarian cancer cells by targeting URI (Xing et al. 2019). However, how miR-598 can be regulated in ovarian cancer cell is still uncertain. Additionally, more downstream regulatory targets of miR-598 in ovarian cancer are expected to be investigated.

Bromodomain-containing protein-4 (BRD4) belongs to the BET family of nuclear proteins that carry 2 bromodomains and an additional ET domain (Zhong et al. 2018). An early study revealed that ectopic expression of mouse BRD4 in mouse fibroblasts and HeLa cells inhibited cell cycle progression from G1 to S (Maruyama et al. 2002), suggesting that BRD4 was associated to cell cyclin and proliferation. Further studies suggested that shRNA or small-molecule inhibitor mediated suppression of BRD4 resulted in robust antileukemic effects in vitro and in vivo, with a terminal myeloid differentiation and an elimination of leukemia stem cells (Zuber et al. 2011), demonstrating that BRD4 is a therapeutic target for acute myeloid leukemia (AML). Importantly, a recent study demonstrated that the activity of BRD4 was necessary for proliferation and survival of two ovarian cell line, and the results of BRD4 inhibition in vitro or in vivo suggested that BRD4 was a potential therapeutic target for ovarian cancer (Baratta et al. 2015). These reports demonstrated that BRD4 is a tumor stimulator for ovarian cancer. The inhibition of BRD4 hosts a promising for the development of ovarian therapeutic agents. In the present study, the bioinformatics analysis revealed that there are putative binding sites of miR-598 in both circCELSR1 (hsa_circ_0063809) and BRD4. Therefore, we speculated that circCELSR1 promoted tumorigenesis of ovarian cancer via sponging miR-598 to increasing BRD4 expression.

## Methods

### Cell culturing

Ovarian cancer cells, including SKOV3, A2780, IGROV1 and CAOV3, and the normal ovarian cell IOSE80 were all obtained from American Type Culture Collection (ATCC, Manassas, VA, USA). SNU119, OVCAR4 and COV362 were obtained from the Cell Bank of the Chinese Academy of Sciences (Shanghai, China). SKOV3, IGROV1, IOSE80, OVCAR4, COV362, SNU119 and CAOV3 are cultured with DMEM (Gibco, Aukland, New Zealand), containing 10% fetal bovine serum (FBS; Gibco, Aukland, New Zealand) supplemented with L-glutamine and antibiotics (1% penicillin/streptomycin). A2780 is cultured with RPMI 1640 medium (Gibco, Aukland, New Zealand), containing 10% fetal bovine serum (FBS; Gibco, Aukland, New Zealand) supplemented with L-glutamine and antibiotics (1% penicillin/streptomycin). Cells were maintained in the 37 °C incubator with 5% CO_2_.

### qRT-PCR

Cells were collected at indicated time points, and total RNA was extracted by Trizol reagent (Invitrogen, Carlsbad, CA, USA) according to manufacturer’s instructions. For reverse transcription, 1–3 μg of RNA, Superscript II (Invitrogen) and chemically synthetic oligo dT were used to generate cDNA. The cDNA was used for qRT-PCR with the SYBR Green Q-PCR mix (Kakara, Dalian, China), according to manufacturer’s instructions. qRT-PCR was carried out in an ABI 7500 thermocycler with fluorescence detection (Applied Biosystems, Carlsbad, CA, USA). A standard qRT-PCR method was used for the amplification, with 94 °C for 2 min as initial denaturation, 40 cycles with 94 °C, 15 s and 60 °C for 1 min. GAPDH and U6 were chose as internal control. Primers are as followed: circCELSR1-forward: 5′-AACTCTCAGGCTGGGACTCA-3′, reverse: 5′-AGTTTCGTTGTGGCTCTGCT-3′; miR-598-forward: 5′-CCGCTACGTCATCGTTGTCA-3′, reverse: 5′-GTCGTATCCAGTGCAGGGTCCGAGGTATTCGCACTGGATACGACTGACGA-3′; BRD4-forward: 5′-GTGGTGCACATCATCCAGTC-3′, reverse: 5′-CCGACTCTGAGGACGAGAAG-3′; U6-forward: 5′-CTCGCTTCGGCAGCACA-3′, reverse: 5′-AACGCTTCACGAATTTGCGT-3′; GAPDH-forward: 5′-TGTGGGCATCAATGGATTTGG-3′, reverse: 5′-ACACCATGTATTCCGGGTCAAT-3′. The 2^−ΔΔCt^ method was used to assess the relative level of mRNA or miRNAs.

### Cell transfection

miR-598 mimics, miR-598 inhibitor and negative control (NC), sh-NC, sh-circCELSR1 were all obtained from Genepharma (Shanghai, China). Control group represented cells without any treatment. For overexpression of circCELSR1, circCELSR1 DNA fragment was fused to pcDNA3.1 vector. For overexpression of BRD4, the gene fragment of BRD4 was fused to pcDNA3.1. The two plasmids are customized ordered from Genscript (Nanjing, China). Lipofectamine 2000 (Invitrogen, Carlsbad, CA, USA) was used for transfection according to the manufacturer’s instructions. The cells were cultured for 48 h after transfection and harvested for the following experiments.

### Dual luciferase reporter assay

Dual luciferase reporter system psiCHECK™ (Fisher Scientific) was used for luciferase assay. The putative circCELSR1 or 3′ untranslated region (3′ UTR) of BRD4 including miR-598 binding region were inserted to the plasmid psiCHECK2. The cells were seeded at 4 × 10^4^ cells/well, and each well was transfected with a total of 400 ng of psiCHECK vector, psiCHECK-BRD4 3’UTR or psiCHECK-circCELSR1 were co-transfected with miR-598 mimics/mimics NC or miR-598 inhibitor/inhibitor NC by lipofectamine2000 (ThermoFisher, USA) 24 h after seeding. Cells were lysed after 24 h of the transfection and the measurement of luciferase activity was performed for each well by Dual-luciferase reporter assay system (Promega, USA).

### Western blotting

Total proteins of thecells were extracted by lysis buffer (Tris-HCl, PH 8.0, 400 mM NaCl, 5 mM EDTA, 1 mM EGTA, 1 mM Na pyrophosphate, 1% Triton X-100, 10% glycerol), with supplement of protease inhibitors (Roche, Indianapolis, IN). The protein concentration was determined by a BCA kit (Piece, Rockford, IL). Then protein was loaded onto 12% SDS-PAGE gel and was electronically transferred to PVDF membranes (Millipore, Billerica, MA). Primary and secondary antibodies were then incubated to the membranes. The HRP-conjugated secondary antibody (1:5000, Sigma-Aldrich) was incubated for 2 h atroom temperature. Protein signals were detected via ECL method. The primary antibodies used in this study includes anti-BRD4 (1:1000, Abcam), anti-c-Myc (1:1000, Abcam), anti-CyclinD1 (1:1000, Abcam), anti-CDK4 (1:1000, Abcam), anti-MMP2 (1:1000, Abcam), anti-MMP9 (1:1000, Abcam), anti-CtIP (1:1000, Abcam), anti-CD274 (1:1000, Abcam), anti-KRAS (1:500, Abcam), anti-E-cadherin (1:10000, Abcam), anti-N-cadherin (1:1000, Abcam), anti-Vimentin (1:3000, Abcam) and anti-GAPDH (1:1000, Abcam). All the primary antibodies were incubated for 4 h at 37 °C.

### Colony formation assay

Cell proliferation of ovarian cancer cells was measured by colony formation assay. Briefly, cells were seeded on 6-well plate and incubated at 37 °C with 5% CO_2_ for colony formation. After 10 days, 10% (v/v) methanol was used to fixcell colonies for 15 min and 0.1% crystal violet (Sigma-Aldrich, St. Louis, Missouri, USA) was used to stain for 30 min for colony visualization. Colony number calculation was performed under microscope (Nikon, Japan).

### Flow cytometry analysis

FITC labeled Annexin V and Propidium iodide (PI) were used for the detection of cell apoptosis of ovarian cancer cells. For Annexin V/PI staining, cells were harvested and resuspended with binding buffer which contains FITC labeled Annexin V and PI. After 15 min’s incubation at room temperature, binding buffer was used to dilute cells and flow cytometer Attune NxT (ThermoFisher) was used for analysis.

### Wound healing assay

Cell migration activity of ovarian cancer cells was measured by wound healing assay. Ovarian cancer cells were seeded into 6-well cell culture plates and cultured to ~ 90% confluence. After 6 h of starvation, a sterile 200 μL pipette tip was used to generate an artificial, homogeneous wound by scratching the monolayer of cells. Images of cells migrating into the wound were captured after 24 h using a microscope.

### Transwell assay

The measuring of cell invasion activity was carried out in a transwell system (Costar, Cambridge, MA, USA). The transwell chamber contains polycarbonate filters with 8-μm pores coated with Matrigel. The cells were seeded on the upper chamber of transwell and fed with serum-free medium. For the lower chamber, 600 μL complete medium was filled. The non-invasive cells were removed with a cotton swabafter 24–48 h incubation, the number of invasive cells were counted following staining with 1% crystal violet, and cells were counted under light microscope.

### Animal model

To determine the in vivo role of circCELSR1 and its downstream pathway, an abdominal cavity metastasis ovarian cancer nude mice model was established. All the experimental protocols were approved by the Ethic Committee of the Third Xiangya Hospital of Central South University. 4-week-old athymic female nude mice were purchased from the SJA Laboratory Animal Co., Ltd. (Hunan, China, *n* = 10). The nude mice were randomly divided into 2 groups with 5 mice in each group. 1 × 10^6^ SKVO3 cells stably expressing sh-circCELSR1 were intraperitoneally injected into nude mice. After 30 days, the mice were sacrificedby cervical dislocation. The nodules on the peritoneal mesothelial surfaces of the abdominal cavity were quantified under low-power magnification using a micrometer.

### Immunohistochemistry

For immunohistochemistry, tumor nodules of the nude mice were collected and were fixed in 10% neutral-buffered formalin and embedded in paraffin, after 5 μm paraffin sections were performed, tissue sections were treated with Peroxidase Blocking Reagent (Bio-rad) and Background Sniper (Biocare Medical, Concord, CA, USA), and then incubated with anti-BRD4 (1:500, Abcam), anti-Ki-67 (1:500, Abcam) or anti-Bax (1:250, Abcam) for 1 h at room temperature. After incubation with MACH 4 Universal HRP Polymer and diaminobenzidine (DAB Biocare Medical), counterstaining with hematoxylin were performed.

### Statistical analysis

The format mean ± standard deviation (SD) values is used for the expression of data. One-way ANOVA with Tukey post hoc test was used for the comparisons between more than two groups. Two-tailed t-test were used for comparisons between two groups. Statistical analysis was performed by using SPSS 10.0. *P* < 0.05 was determined to be statistically significant.

## Results

### Gene expression of circCELSR1, miR-598 and BRD4 in ovarian cancer cells

**Fig. 1:**
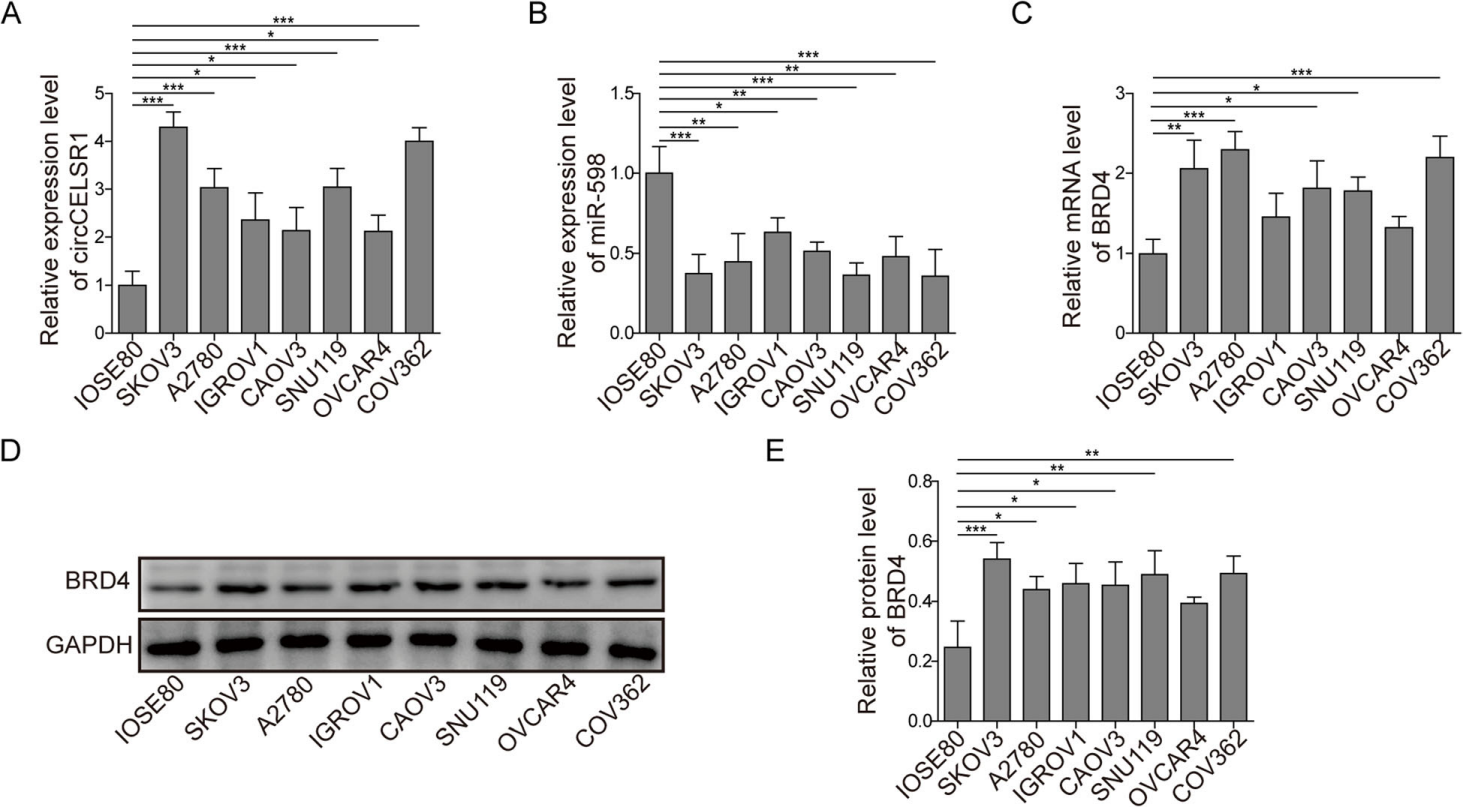
The expression level of circCELSR1, miR-598 and BRD4 in ovarian cancer cells. The level of circCELSR1 (**a**), miR-598 (**b**) and BRD4 (**c**) in IOSE80, SKOV3, A2780, IGROV1, CAOV3, SNU119, OVCAR4 and COV362 were measured by qRT-PCR. The protein level of BRD4 in IOSE80, SKOV3, A2780, IGROV1 and CAOV3, SNU119, OVCAR4 and COV362 were measured by Western blotting (**d**), and Western blotting results were quantified (**e**). All the results were shown as mean ± SD (*n* = 3). * *p* < 0.05 and ** *p* < 0.01

To investigate the function of circCELSR1 and the putative relative genes miR-598 and BRD4 in regulating ovarian cancer, first we examined the expression of these genes in ovarian cancer cells. Gene expression in seven kinds of ovarian cancer cells, including SKOV3, A2780, IGROV1, CAOV3, SNU119, OVCAR4 and COV362 were examined. The ovarian cell IOSE80 is considered as a normal control of a subset of high-grade serous ovarian cancer, and we used it as normal control in the current study. As shown in Fig. 1a, the expression level of circCELSR1 was notably up-regulated in the seven ovarian cancer cells, compared to the normal ovarian cell line IOSE80. The relative increase of circCELSR1 expression was about 4.25, 3.11, 2.46, 2.17, 2.98, 2.25 and 4.06 folds in cell line SKOV3, A2780, IGROV1, CAOV3, SNU119, OVCAR4 and COV362, respectively. As shown in Fig. 1b and c, miR-598 expression was decreased and BRD4 was increased in the ovarian cancer cells. The relative decrease of miR-598 was about 0.41, 0.47, 0.67, 0.58, 0.40, 0.51 and 0.39 fold in cell line SKOV3, A2780, IGROV1, CAOV3, SNU119, OVCAR4 and COV362, respectively; the relative increase of BRD4 was about 2.05, 2.25, 1.43, 1.82, 1.79, 1.35 and 2.17 folds in cell line SKOV3, A2780, IGROV1, CAOV3, SNU119, OVCAR4 and COV362, respectively. Additionally, the protein level of BRD4 in ovarian cancer cells was promoted significantly, as confirmed by Western blotting (Fig. 1d and e). The quantitative value of BRD4 protein relative to GAPDH was about 0.24, 0.62, 0.43, 0.46, 0.45, 0.52, 0.41, 0.52 in IOSE80, SKOV3, A2780, IGROV1, CAOV3, SNU119, OVCAR4 and COV362, respectively. Taken together, the expression of circCELSR1 and BRD4 were promoted, however, miR-598 expression was suppressed in ovarian cancer cells.

### circCELSR1 sponges miR-598 to stimulate BRD4 gene expression

**Fig. 2:**
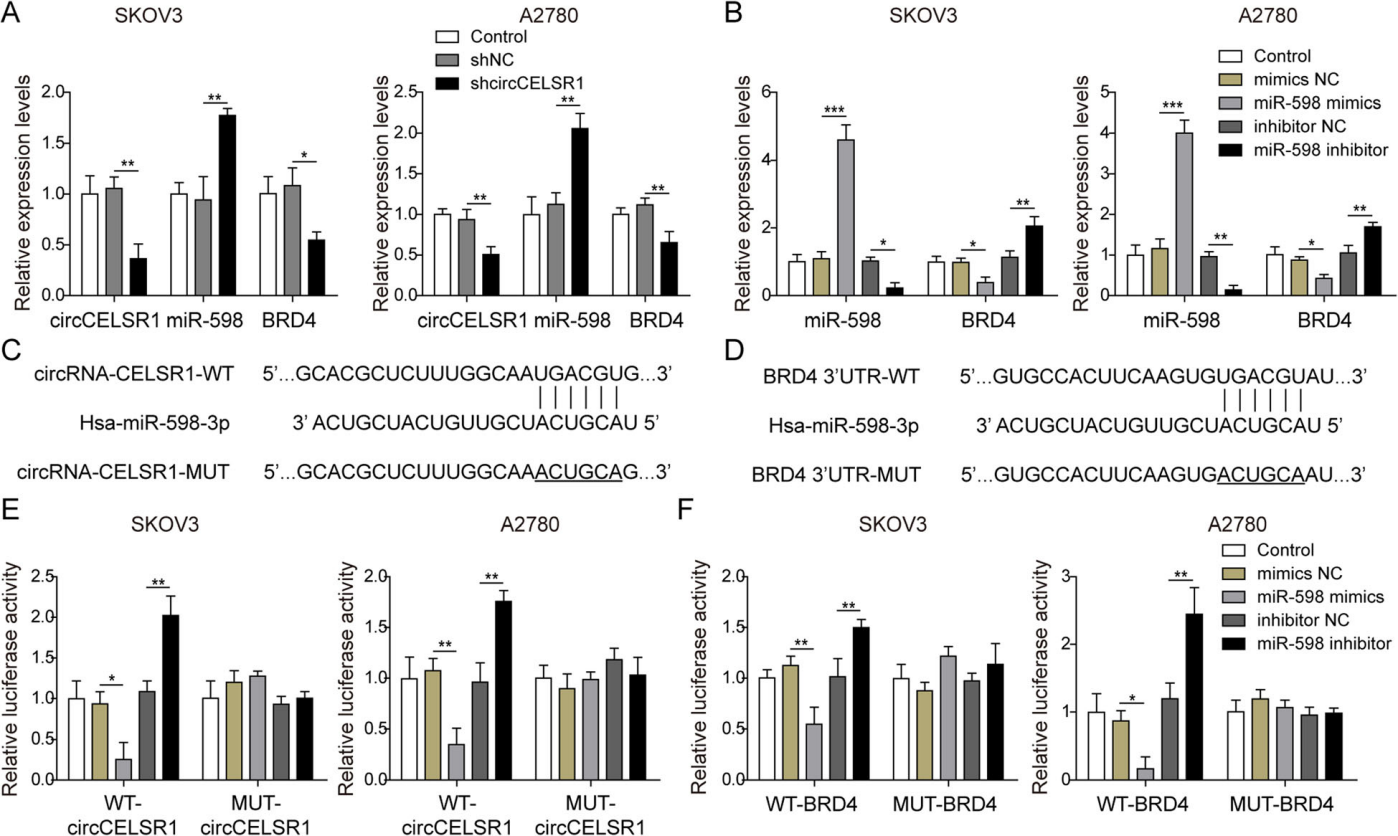
circCELSR1 sponged miR-598 to stimulate BRD4 gene expression in SKOV3 and A2780 cells. **a** Gene expression of circCELSR1, miR-598 and BRD4 were detected by qRT-PCR in SKOV3 and A2780 cells transfected with sh-circCELSR1 or shNC. Control group represented cells without any treatment. **b** Gene expression of miR-598 and BRD4 were detected by qRT-PCR in SKOV3 and A2780 cells transfected with miR-598 mimics and miR-598 inhibitor. Control group represented cells without any treatment. Bioinformatics prediction of the binding site between circCELSR1 and miR-598 (**c**) and between miR-598 and BRD4 3′-UTR (**d**). Luciferase activity assay was performed after co-transfection with circCELSR1 reporter plasmid and miR-598 into SKOV3 and A2780 cells. **f** Luciferase activity assay was performed after co-transfection with BRD4 reporter plasmid and miR-598 into SKOV3 and A2780 cells. All the results were shown as mean ± SD (*n* = 3). * *p* < 0.05 and ** *p* < 0.01

To investigate the regulatory relationship between circCELSR1, miR-598 and BRD4, knockdown of circCELSR1 was performed in ovarian cancer cells. As shown in Fig. 2a, compared to the control which transfected with sh-NC, the group of ovarian cancer cells transfected with sh-circCELSR1 resulted in 68 and 49% decrease of circCELSR1 in SKOV3 and A2780 cells, respectively. miR-598 were up-regulated by 1.76 and 2.19 times, respectively, and the BRD4 level was reduced by 1.82 and 1.54 times in both SKOV3 and A2780 cells (Fig. 2a). These results suggested that circCELSR1 might suppress miR-598 expression but promote BRD4 expression. Furthermore, the transfection of miR-598 mimics resulted in up-regulation of miR-598 by 4.73 and 3.96 times in SKOV3 and A2780 cells, respectively, while BRD4 was decreased by 2.56 and 2.32 times in both SKOV3 and A2780 cells (Fig. 2b). The inhibition of miR-598 in SKOV3 and A2780 mediated by a transfection of miR-598 inhibitor led to notable reduction of miR-598 (reduced by 4.35 and 7.14 times respectively) and significant promotion of BRD4 (increased by 2.06 and 1.73 times respectively) in SKOV3 and A2780 cells (Fig. 2b), suggesting that miR-598 inhibited BRD4 gene expression. Moreover, bioinformatics analysis indicated that there was a binding site of circCELSR1 to miR-598, as shown in Fig. 2c, and a binding site of miR-598 to the BRD4 3′-UTR, as shown in Fig. 2d. The results of luciferase assays indicated that the circCELSR1 and BRD4 3′-UTR wildtype mediated luciferase activity was suppressed by miR-598 mimics, while was promoted by miR-598 inhibitor in bothSKOV3 and A2780 cells (Fig. 2e and f). Moreover, circCELSR1 and BRD4 mutant mediated luciferase was not affected by miR-598 mimics or miR-598 inhibitor (Fig. 2e and f). These results clearly suggested that circCELSR1 could promote BRD4 expression via sponging miR-598 in ovarian cancer cells.

### Knockdown of circCELSR1 inhibits proliferation and promotes apoptosis of ovarian cancer cells by targeting miR-598

**Fig. 3:**
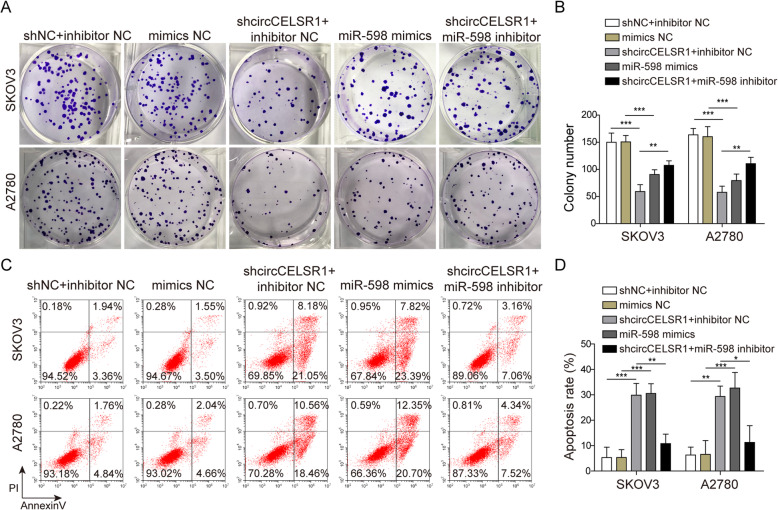
Knockdown of circCELSR1 inhibited proliferation and promoted apoptosis via miR-598 in SKOV3 and A2780 cells. After SKOV3 and A2780 cells transfected with sh-circCELSR1, miR-598 mimics or miR-598 inhibitor, (**a**) Colony formation assay to detect SKOV3 and A2780 cells colony-forming ability. **b** The colony formation number was quantified by Image J. **c** The SKOV3 and A2780 cells apoptotic rate was determined by flow cytometry. **d** Quantitative analysis of cell apoptotic rate. All the results were shown as mean ± SD (*n* = 3). * *p* < 0.05 and ** *p* < 0.01

To evaluate the function of circCELSR1 and miR-598 in the regulation of ovarian cancer cell tumorigenic activity, knockdown of circRNA-CELSR1 and transfection of miR-598 mimics or miR-598 inhibitor were performed. Colony formation assays demonstrated that cell proliferation was inhibited by the knockdown of circCELSR1 or miR-598 mimics, suggesting that circCELSR1was a positive regulator, and miR-598 was a suppressor for ovarian cancer (Fig. 3a and b). Moreover, miR-598 inhibitor was found to reverse the suppression of cell proliferation mediated by knockdown of circCELSR1, suggesting that circCELSR1 regulated ovarian cancer cell proliferation was miR-598 dependent. Additionally, as shown by flow cytometry results in Fig. 3c and d, knockdown of circCELSR1 and overexpression of miR-598 could promote cell apoptosis, but inhibition of miR-598 significantly reversed the above change induced by circCELRS1 knockdown in SKOV3 and A2780. Taken together, knockdown of circCELSR1 could inhibit cell proliferation and promote cell apoptosis by targeting miR-598 in ovarian cancer.

### Knockdown of circCELSR1 suppresses migration and invasion of ovarian cancer cells by targeting miR-598

**Fig. 4:**
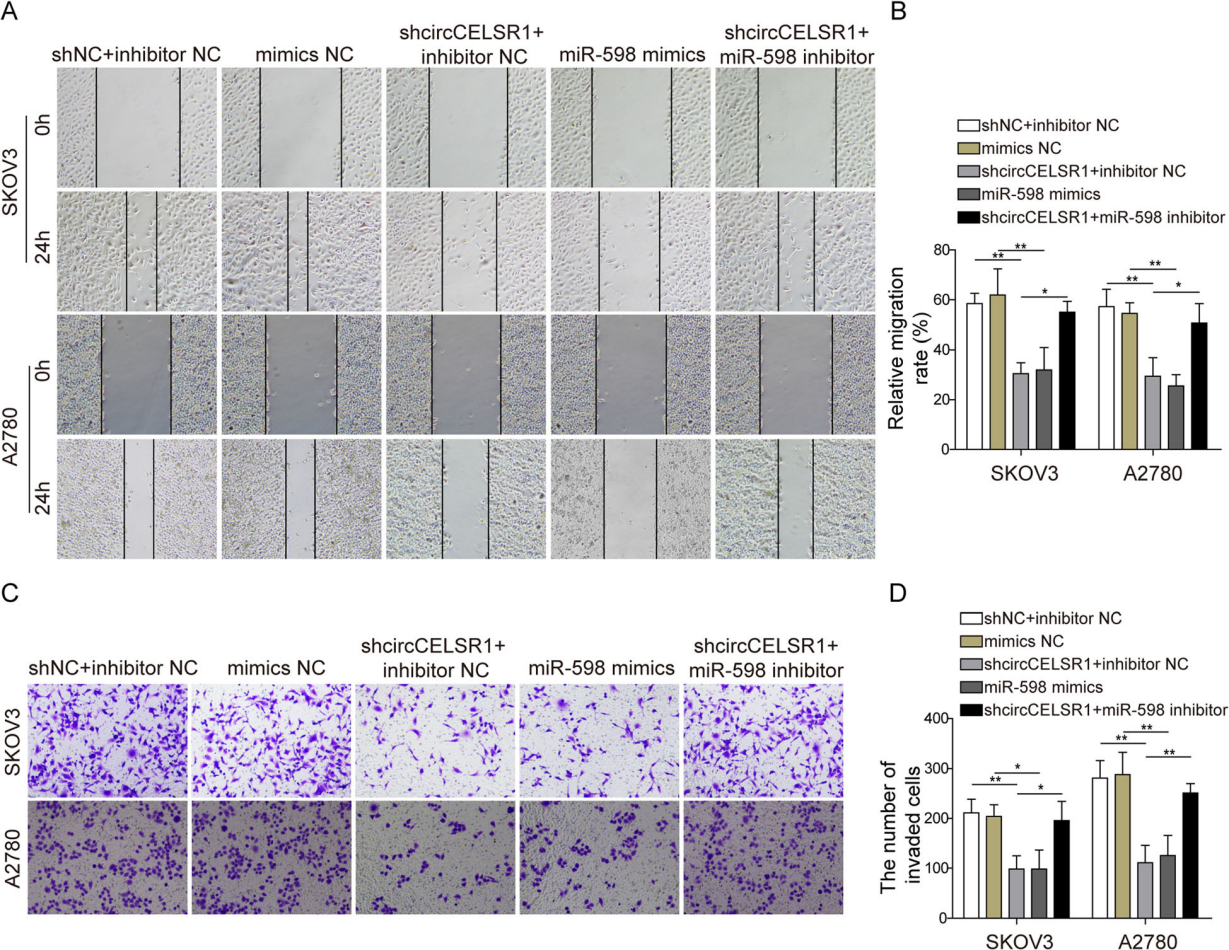
Knockdown of circCELSR1 repressed migration and invasion of ovarian cancer cells via targeting miR-598. Cell migration of SKOV3 and A2780 were measured by wound healing assays (**a**) and quantification of relative migration rate (**b**) in SKOV3 and A2780 cells transfected with sh-circCELSR1, miR-598 mimics or miR-598 inhibitor. Cell invasion of SKOV3 and A2780 were measured by transwell system mediated cell invasion assays (**c**) and quantification of number of invaded cells (**d**). All the results were shown as mean ± SD (*n* = 3). * *p* < 0.05 and ** *p* < 0.01

To evaluate the function of circCELSR1 and miR-598 in regulating cell migration and invasion of ovarian cancer cells, knockdown of circCELSR1 and transfection of miR-598 mimics or miR-598 inhibitor were performed. As shown by wound healing assay results in Fig. 4a and b, for ovarian cancer cell SKOV3 and A2780, cell migration was inhibited by circCELSR1 knockdown or transfection of miR-598 mimics, and miR-598 inhibitor resulted in a rescue of cell migration. Next, the regulation of cell invasion activity by circCELSR1 and miR-598 were evaluated via transwell cell invasion assay. As shown in Fig. 4c and d, knockdown of circCELSR1 or miR-598 mimics transfection led to suppression of cell invasion, but the cell invasion activity was rescued by the transfection of miR-598 inhibitor. These results suggested that circSELSR1 promotes cell migration and invasion of ovarian cancer cells by suppressing miR-598.

### Knockdown of circCELSR1 represses BRD4-mediated proliferation/migration related signaling by targeting miR-598

**Fig. 5:**
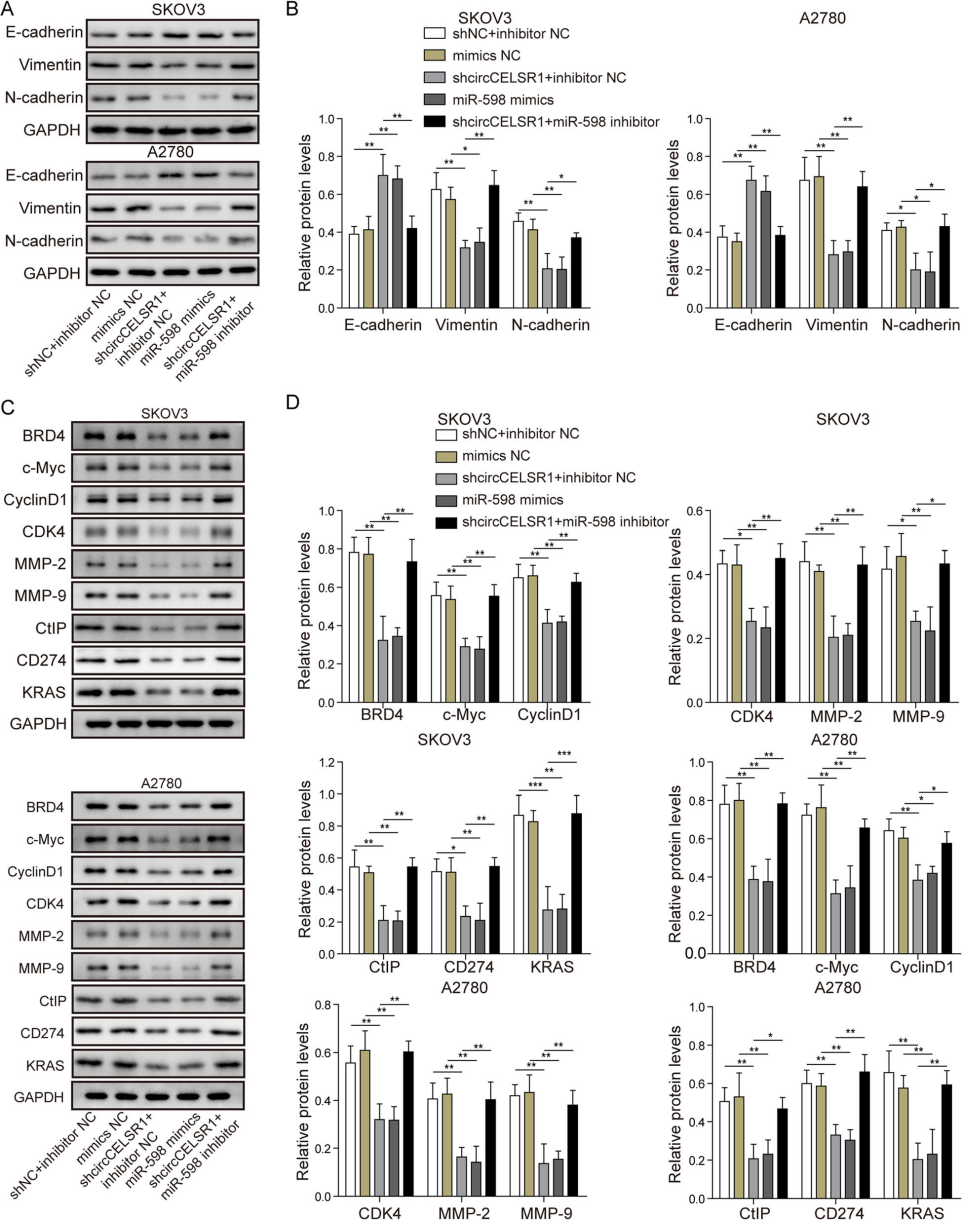
Knockdown of circCELSR1 inhibited the expression of BRD4 and EMT, proliferation/migration related genes via targeting miR-598. Protein level of E-cadherin, Vimentin, N-Cadherin (**a**), were measured by Western blotting, the quantification of relative protein level (**b**) in SKOV3 and A2780 cells that transfected with sh-circCELSR1, miR-598 mimics or miR-598 inhibitor. Protein level of BRD4, c-Myc, CyclinD1, CDK4, MMP-2, MMP-9, Ctlp, CD274 and KRAS were measured by Western blotting (**c**) and the quantification of relative protein level (**d**) in SKOV3 and A2780 cells transfected with sh-circCELSR1, miR-598 mimics or miR-598 inhibitor. All the results were shown as mean ± SD (*n* = 3). * *p* < 0.05 and ** *p* < 0.01

In this section of study, we examined the effects of circCELSR1 and miR-598 on BRD4 and its mediated proliferation/migration-associated proteins by Western blotting. Considering that miR-598 was previously found to regulate EMT, here we examined whether circCELSR1 regulates EMT via miR-598. As shown in Fig. 5a and b, our results indicated that, knockdown of circCELSR1 or overexpression of miR-598 promoted the expression of E-cadherin, and suppressed the expression of Vimentin and N-cadherin, suggesting that circCELSR1 regulated EMT via targeting miR-598. As shown in Fig. 5c and d, in both SKOV3 and A2780, the protein level of BRD4 and proliferation related proteins c-Myc, CyclinD1 and CDK4, as well as the migration related proteins MMP-2 and MMP-9 were all found to be decreased by circCELSR1 knockdown or miR-598 mimics. Furthermore, miR-598 inhibitor could reverse the effects caused by circCELSR1 inhibition. These results suggested that circCELSR1 promoted the expression of BRD4 and activated proliferation/migration related signaling by targeting miR-598. Additionally, the protein expression of CtIP, CD274, and KRAS, which were proven to be regulated by BRD4, were also evaluated. Our results indicated that, knockdown of circCELSR1 or overexpression of miR-598 resulted in suppression of CtIP, CD274, and KRAS protein expression. Additionally, knockdown of miR-598 reversed circCELSR1 knockdown mediated suppression of CtIP, CD274, and KRAS protein expression (Fig. 5c and d). These results suggested that circCELSR1 promoted the expression of BRD4 to stimulate the expression of CtIP, CD274, and KRAS. Considering that the native expression level of BRD4 is diverse in different cell lines, we evaluated the effects of circCELSR1 in regulating BRD4 in different cell lines. As shown in Fig. S1, SKOV3 cell line, which has high expression of BRD4, and OVCAR4 cell line, which has low expression of BRD4, were used to examine the modulation of BRD4 expression by circCELSR1 overexpression. Our results indicated that overexpression of circCELSR1 led to significant promotion of BRD4 expression in both SKOV3 and OVCAR4. Moreover, the promotion of BRD4 by circCELSR1 was found to be more significant in OVCAR4 (Fig. S1).

### Overexpression of BRD4 reverses shcircCELSR1 mediatied effects of proliferation, migration, invasion and apoptosis

**Fig. 6:**
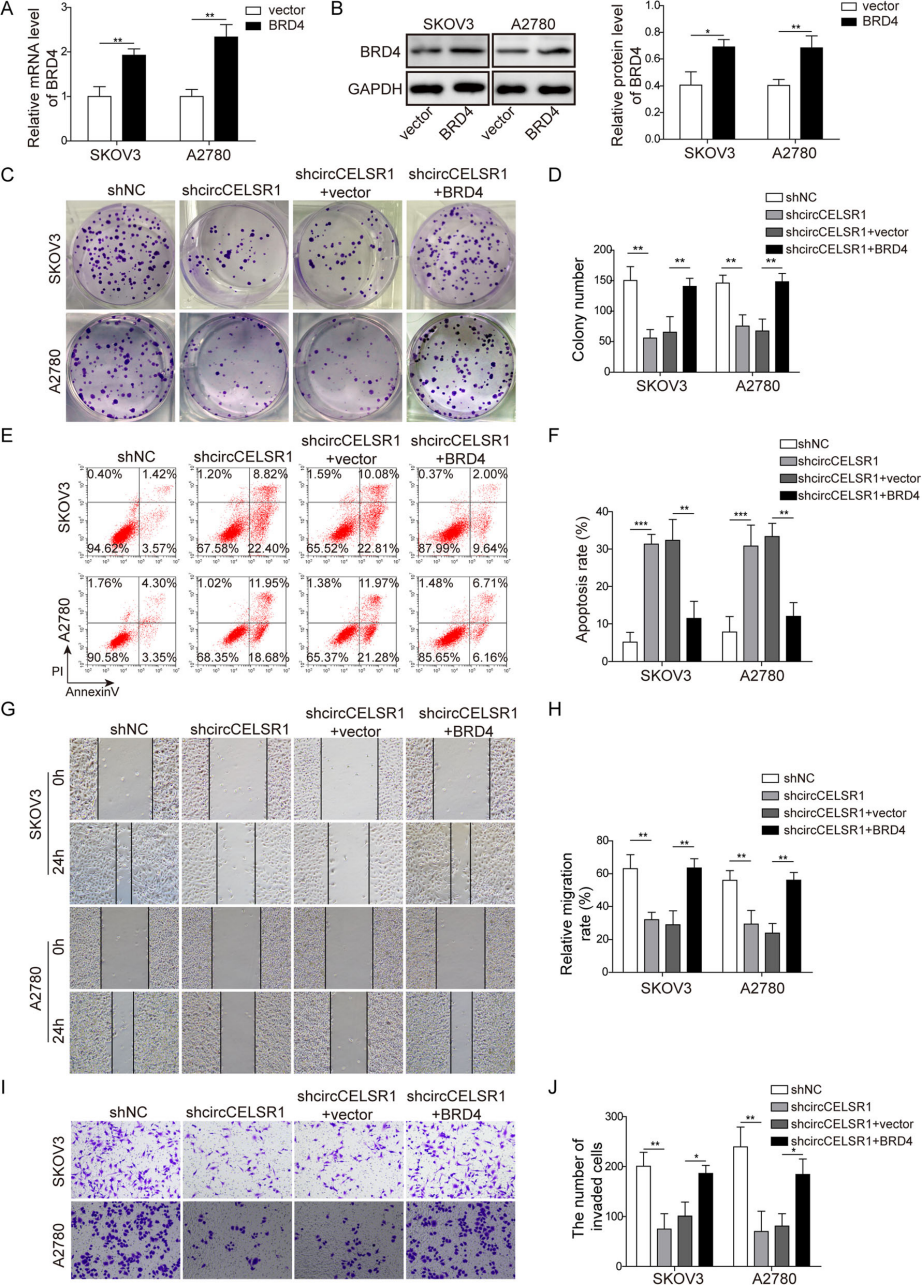
circCELSR1 regulated ovarian cancer cell activities via BRD4. **a** SKOV3 and OVCAR4 cells were transfected with pcDNA3.1-BRD4 and mRNA level was evaluated by qRT-PCR. **b** SKOV3 and A2780 were transfected with pcDNA3.1-BRD4 and protein level of BRD4 were evaluated by Western blotting. **c** SKOV3 and A2780 were transfected with shcircCELSR1 or/and pcDNA3.1-BRD4, and colony formation assay were performed, and (**d**) quantification of colony formation was shown. **e** SKOV3 and A2780 were transfected with shcircCELSR1 or/and pcDNA3.1-BRD4, and cell apoptosis were evaluated by FACS, and (**f**) the quantification of apoptosis rate was shown. **g** SKOV3 and A2780 were transfected with shcircCELSR1 or/and pcDNA3.1-BRD4, and cell migration were measured by wound healing assay, and (**h**) quantification of cell migration was shown. **i** SKOV3 and A2780 were transfected with shcircCELSR1 or/and pcDNA3.1-BRD4, and cell invasion was measured by invasion assay, and (**j**) invasion rate was shown. All the results were shown as mean ± SD (*n* = 3). * *p* < 0.05 and ** *p* < 0.01

To further confirm the putative regulatory mechanism circCELSR1/miR-598/BRD4 in the regulation of ovarian cancer cell activities, a rescue experiment was performed. As shown in Fig. 6a and b, in ovarian cancer cell line SKOV3 and A2780, the mRNA and protein level of BRD4 were promoted by the transfection of pcDNA3.1-BRD4. In Fig. 6c and d, our results indicated that the knockdown of circCELSR1 in the two ovarian cancer cell lines led to reduction of colony numbers, however, the colony numbers were rescued by overexpression of BRD4. Furthermore, shcircCELSR1 promoted cell apoptosis rates, and this were reversed by BRD4 overexpression (Fig. 6e and f). To ascertain the effect of cell migration, wound healing assay was performed. circCELSR1 knockdown in this two ovarian cancer cell lines resulted in suppression of cell migration, and BRD4 overexpression reversed this effect (Fig. 6g and h). For cell invasion, the results of transwell mediated invasion assay suggested that circCELSR1 knockdown suppressed cell invasion, and similarly could be reversed by BRD4 overexpression (Fig. 6i and j). Collectively, these results demonstrated that overexpression of BRD4 reverses shcircSELSR1 mediatied suppression of tumorigenic activity of ovarian cancer cell line SKOV3 and A2780.

### Knockdown of circCELSR1 attenuates ovarian cancer growth and metastasis, promotes apoptosis by regulating miR-598/BRD4 axis in nude mice

**Fig. 7:**
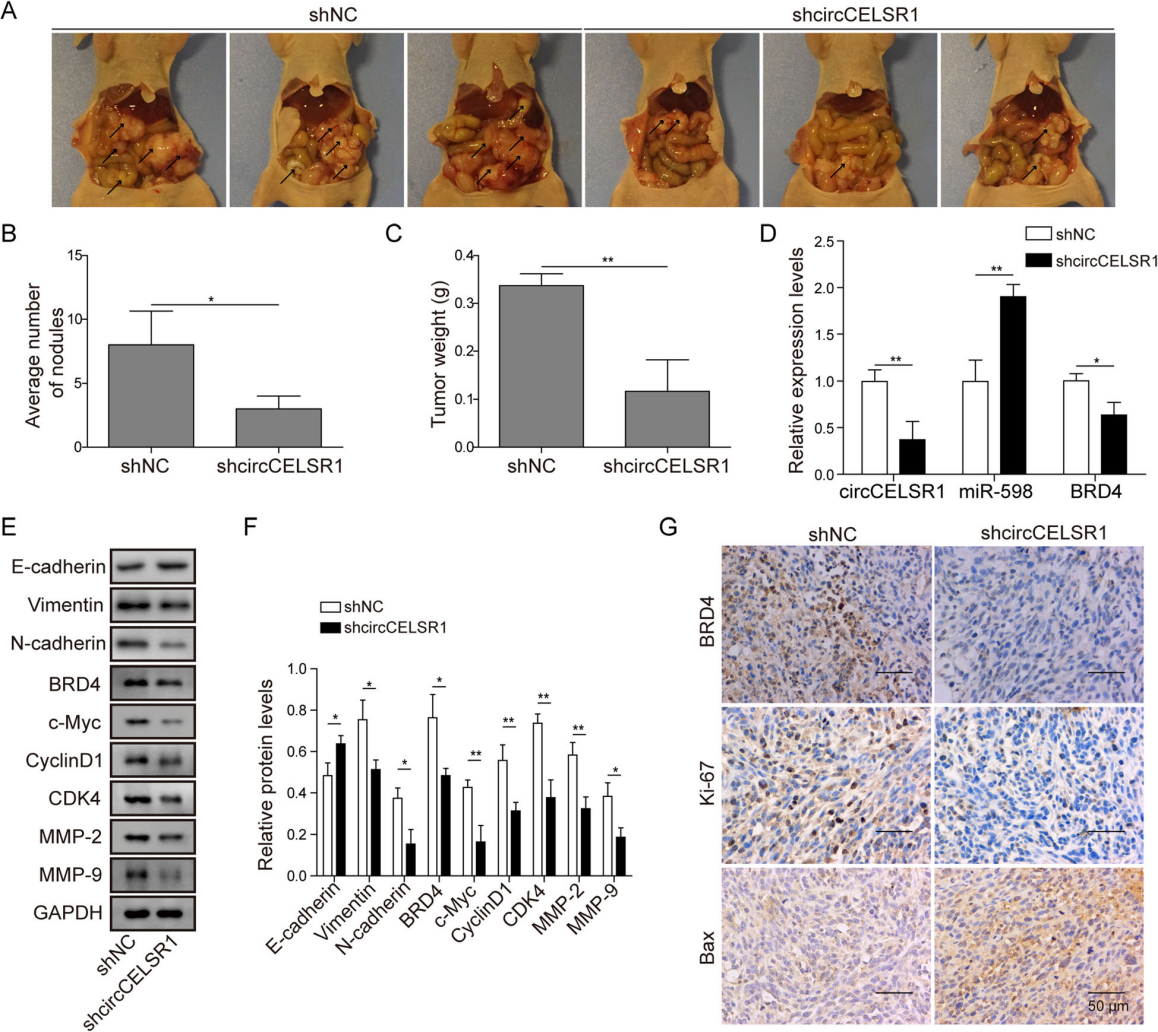
circCELSR1/miR-598/BRD4 axis regulated ovarian cancer progression in vivo. **a** Pictures showed the metastatic tumors in nude mice. **b** Quantification of average number of tumor nodules and (**c**) tumor weight were shown. **d** The gene expression of circCELSR1, miR-598 and BRD4 in xenografted tumors was measured by qRT-PCR. **e** Protein level of E-cadherin, Vimentin, N-cadherin, BRD4, c-Myc, CyclinD1, CDK4, MMP-2 and MMP-9 in xenografted tumors were measured by Western blotting, and (**f**) quantification of protein level was shown. **g** The protein level of BRD4, Ki-67 and Bax in tumor sections was measured by immunohistochemistry. All the results were shown as mean ± SD (*n* = 3). * *p* < 0.05 and ** *p* < 0.01

To evaluate the in vivo function of circCELSR1 in regulating ovarian cancer metastasis, an abdominal cavity metastasis model in nude mice was established. As shown in Fig. 7a, the pictures showed the metastatic ovarian tumor tissues in abdominal cavity were found to be reduced significantly in the nude mice xenografted with sh-circCELSR1 ovarian cells. This was validated by the quantification of average nodule number and tumor weigh (Fig. 7b and c). Next, the putative circCELSR1/miR-598/BRD4 regulatory axis was examined in the in vivo ovarian tumors. As shown in Fig. 7d, the level of circCELSR1 and BRD4 were found to be suppressed significantly, oppositely, the level of miR-598 was promoted, suggesting that the knockdown of circCELSR1 resulted in a promotion of miR-598 and a suppression of BRD4 in vivo. Next, the level of BRD4 and proliferation/migration/EMT related proteins were evaluated. Western blotting results indicated that the protein level of BRD4, c-Myc, CyclinD1, CDK4, MMP-2, MMP-9, Vimentin and N-cadherin were all decreased while E-cadherin was increased in the tumor with circCELSR1 knockdown (Fig. 7e and f). These results suggested that knockdown of circCELSR1 suppressed the expression of BRD4 and the proliferation/migration proteins in vivo. Immunohistochemistry results demonstrated that the level of BRD4 and Ki-67 were suppressed by circCELSR1 knockdown, suggesting that circCELSR1 promoted BRD4 and tumor cell proliferation in vivo (Fig. 7g). Additionally, considering that circCELSR1 exerts a regulatory role for cellular apoptosis of ovarian cells, an evaluation of apoptotic marker Bax was performed by IHC in ovarian cancer xenograft tumor. As shown in Fig. 7g, Our results indicated that konckdown of circCELSR1 promoted the expression of Bax in the tumor tissues of ovarian xenograft nude mice. Taken together, our results from the abdominal cavity metastasis model demonstrated that circCELSR1 promotes ovarian cancer progression by stimulating BRD4 expression.

## Discussion

Due to the difficulty of early diagnosis and relative low effectivity for therapeutic agents, the mortality of ovarian cancer is still high worldwide (Torre et al. 2018). A group of therapeutic markers have been identified, for example tumor associate antigens (TAA) CA125 (Patankar et al. 2005) and the immune checkpoint molecules, including PD1/PD-L1 (Matsuzaki et al. 2010) and CTLA-4 (Gooden and van Hall 2012), etc. However, the effective drugs available in clinical market are still limited. Deeper insight into the molecular mechanism is necessary, and more therapeutic targets are still to be identified. In the present study, we identified that circCELSR1 promotes BRD4 expression and ovarian cancer progression via sponging miR-598. This new mechanism may elicit deeper insight for understanding ovarian cancer and the development of therapeutic methods.

circRNAs are found to be involved in the regulation of ovarian cancer progression recently, as summarized by the up-to date published reviews (Liu et al. 2019). According to the publications, we found that circRNAs affects ovarian cancer progression majorly by modulating cell proliferation and metastasis (migration and invasion) (Zhao et al. 2019), EMT (Zhang et al. 2019) and apoptosis (Chen et al. 2019). From the view of molecular mechanism, most of the studies claimed the circRNAs which they are studying affects ovarian cancer cell activities through ceRNA mechanism. For example, circCSPP1 sponged miR-1236-3p to promote proliferation, migration and invasion of ovarian cancer cells (Li et al. 2019). circEPSTI1 regulated ovarian cancer progression by sponging miR-942 (Xie et al. 2019).CircITCHsuppressed ovarian carcinoma by sponging miR-145 to regulating RASA1 signaling (Hu et al. 2018). circCELSR1 was a recently identified circular RNA, and its exact role in cancer progression is still to be elucidated. There were only two reports to reveal that circCELSR1 is relevant to the regulation of ovarian cancer (Ning et al. 2018; Zhang et al. 2020), however, the function and mechanism of circCELSR1 is still unknown. In the present study, we identified that the expression of circCELSR1 was up-regulated in ovarian cancer cells, and knockdown of circCELSR1 resulted in a suppression of cell proliferation, migration and invasion, moreover, a promotion of apoptosis. Finally, the results from in vivo abdominal cavity metastasis model in nude mice indicated that knockdown of circCELSR1 suppressed ovarian cancer proliferation and metastasis. Taken together, our in vitro and in vivo evidence demonstrated that circCELSR1 promotes ovarian cancer progression.

miRNAs have been proven to be crucial in ovarian cancer development (Deb et al. 2018). MiRNAs may be a tumor suppressor or promoter for regulating ovarian cancer. For example, miR-654-5p was identified to be a suppressor for ovarian cancer by targeting to different genes that included in MYC, WNT and AKT pathways (Majem et al. 2019). miR-450a was found to be tumor suppressor of ovarian cancer by targeting energy metabolism associated genes (Muys et al. 2019). miR-9 was found to promote EMT and metastasis of ovarian cancer (Sui et al. 2019). miR-1251-5p was found to promote carcinogenesis and autophagy of ovarian cancer cell by targeting tumor suppressor TBCC (Shao et al. 2019). It was reported that miR-598 was found to play role is the regulation of different cancers (Tong et al. 2018; Liu et al. 2018). Recent studies indicated that miR-598 might suppress ovarian cancer progression by targeting URI (Xing et al. 2019). However, the molecular mechanism of this regulation needs deeper insight. Our results suggest that miR-598 was significantly down-regulated in ovarian cancer cells, and circCELSR1 acted as ceRNA to sponge miR-598 and inhibited its function. miR-598 reversed circCELSR1 mediated ovarian cancer tumorigenic activity, suggesting that circCELSR1/miR-598 pathway was critical in regulating ovarian cancer. Our results are consistent with the previous reports and proved that miR-598 was a tumor suppressor for ovarian cancer. Moreover, here we found that miR-598 is suppressed by circCELSR1, thus the circCELSR1/miR-598 pathway facilitates ovarian cancer tumor progression.

As reported, the protein BRD4 was proven to be necessary for proliferation and survival of two ovarian cell line, and the results of BRD4 inhibition in vitro or in vivo suggested that BRD4 was a potential therapeutic target for ovarian cancer (Baratta et al. 2015). In cancer development, BRD4 was a stimulator for the expression of cell proliferation related genes, such as c-Myc, CyclinD1 and CDK4 (Segura et al. 2013; Xiang et al. 2018), as well as cell migration related genes, such as MMP-2 and MMP-9 (Wang et al. 2015). In this study, we found that the expression of BRD4 was promoted in ovarian cancer cells, which was consistent with previous reports. miR-598 could decrease BRD4 expression by directly binding to its 3’UTR, and circCELSR1 promoted the expression of BRD4 by targeting miR-598. Additionally, our in vitro and in vivo results demonstrated that circCELSR1/miR-598 pathway regulated BRD4 downstream proliferation/migration related genes, suggesting that circCELSR1/miR-598 pathway regulates ovarian cancer cell proliferation and migration through BRD4. Taken together, circCELSR1 sponges miR-598 to promote gene expression of BRD4 and facilitates ovarian cancer progression.

## Conclusions

Collectively, we identified that circCELSR1/miR-598/BRD4 pathway is a new regulatory mechanism in ovarian cancer progression. circCELSR1 may be a new therapeutic target for the treatment of ovarian cancer. The design of siRNA or shRNA nucleic acid drugs targeting circCELSR1 may be a new way for the treatment of ovarian cancer. Moreover, circCELSR1 may be a novel biomarker for the molecular diagnosis of ovarian cancer.

## Supplementary information

**Additional file 1: Figure S1.** Comparision of the regulation of BRD4 by circCELSR1 in SKVO3 and OVCAR4. (A) SKVO3 and OVCAR4 were transfected with pcDNA3.1-circCELSR1 for circCELSR1 overexpression, and the circCELSR1 expression level was measured by qRT-PCR. (B) SKVO3 and OVCAR4 were transfected with pcDNA3.1-circCELSR1 for circCELSR1 overexpression, and the protein level of BRD4 were measured by Western blotting, and (C) shows the quantification of Western blotting results. All the results were shown as mean ± SD (*n* = 3). * *p* < 0.05 and ** *p* < 0.01.

## Data Availability

All data generated or analysed during this study are included in this published article.

## Abbreviations

EMT: Epithelial mesenchymal transition
BRD4: Bromodomain-containing protein-4
AML: Acute myeloid leukemia
TAA: Tumor associate antigens
