# Supplementary figures and images for: circCELSR1 facilitates ovarian cancer proliferation and metastasis by sponging miR-598 to activate BRD4 signals
Source: Mol Med. 2020 Jul 8;26:70. doi: 10.1186/s10020-020-00194-y (PMC7346459; doi:10.1186/s10020-020-00194-y)

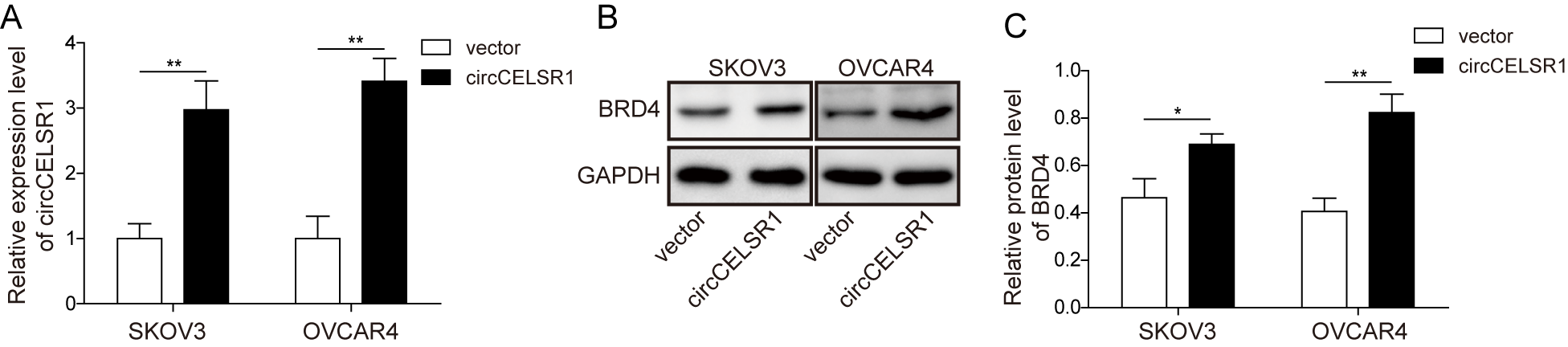

Supplement: Supplementary file 1 — Additional file 1: Figure S1. Comparision of the regulation of BRD4 by circCELSR1 in SKVO3 and OVCAR4. (A) SKVO3 and OVCAR4 were transfected with pcDNA3.1-circCELSR1 for circCELSR1 overexpression, and the circCELSR1 expression level was measured by qRT-PCR. (B) SKVO3 and OVCAR4 were transfected with pcDNA3.1-circCELSR1 for circCELSR1 overexpression, and the protein level of BRD4 were measured by Western blotting, and (C) shows the quantification of Western blotting results. All the results were shown as mean ± SD (n = 3). * p < 0.05 and ** p < 0.01. [file 10020_2020_194_MOESM1_ESM.tif]
